# Evaluation of subclinical cardiovascular disease by carotid intima media thickness, epicardial adipose tissue thickness, serum endocan, and nesfatin-1 levels in patients with primary hyperparathyroidism

**DOI:** 10.55730/1300-0144.5405

**Published:** 2022-04-02

**Authors:** Muhammet KOCABAŞ, Yakup ALSANCAK, Mustafa CAN, İlker ÇORDAN, Hatice ÇALIŞKAN BURGUCU, Melia KARAKÖSE, Fatma Hümeyra YERLİKAYA, Mustafa KULAKSIZOĞLU, Feridun KARAKURT

**Affiliations:** 1 Division of Endocrinology and Metabolism, Department of Internal Medicine, Faculty of Medicine, Necmettin Erbakan University, Konya, Turkey; 2 Department of Cardiology, Faculty of Medicine, Necmettin Erbakan University, Konya, Turkey; 3 Department of Endocrinology and Metabolism, Konya City Hospital, Konya, Turkey; 4 Department of Biochemistry, Faculty of Medicine, Selçuk University, Konya, Turkey

**Keywords:** Primary hyperparathyroidism, carotid intima media thickness, epicardial adipose tissue thickness, endocan, nesfatin-1, cardiovascular disease risk

## Abstract

**Background/aim:**

Data on the presence and extent of cardiovascular disease (CVD) risk in primary hyperparathyroidism (PHPT) are conflicting. In our study, we aimed to investigate the increased CVD risk in patients with PHPT by carotid intima-media thickness (CIMT), epicardial adipose tissue (EAT) thickness, and serum levels of endocan and nesfatin-1.

**Materials and methods:**

Patients with PHPT (n = 44) and age- and sex-matched healthy control subjects (n = 40) were enrolled in this study. Demographic data of the participants were questioned. Serum endocan and nesfatin-1 concentrations were assessed using commercially available ELISA kits. Noninvasive measurements of CIMT and EAT thickness were made with high-resolution ultrasonography and B-mode echocardiography.

**Results:**

There was no statistically significant difference in serum endocan and nesfatin-1 levels and EAT thickness in the PHPT group compared to controls. CIMT was statistically significantly higher in the PHPT group compared to controls (p = 0.001). A negative correlation was found between PTH and low-density lipoprotein cholesterol level (p = 0.001) but no significant relationship was found between other parameters.

**Conclusion:**

We found that CIMT is increased in patients with PHPT and consequently, CVD risk is high in these patients. More comprehensive studies are needed to identify other markers that predict increased CVD risk in patients with PHPT.

## 1. Introduction

Primary hyperparathyroidism (PHPT) is the most common cause of hypercalcemia detected in outpatients [1] and is characterized by hypercalcemia and high or inappropriately normal parathyroid hormone (PTH) levels. PHPT is known to be associated with cardiac and metabolic morbidities such as abnormal cardiac calcification, reversible left ventricular hypertrophy, impaired glucose tolerance, diabetes mellitus (DM), hypertension (HT), dyslipidemia and obesity [2 – 9].

Endothelial dysfunction is considered an early feature of developing atherosclerosis and is characterized by the promotion of vascular dysfunction and progression of atherosclerosis [10]. Carotid intima media thickness (CIMT) is known as a marker of subclinical atherosclerosis and has been associated with cardiovascular events and mortality [11]. Epicardial adipose tissue (EAT) is located between the outer layer of the myocardium and the inner layer of the pericardium. EAT is metabolically active and can cause inflammation and dysfunction in the myocardium by secreting proatherogenic and proinflammatory cytokines as well as reactive oxygen radicals.

Endocan (endothelial-cell-specific molecule-1) is a soluble proteoglycan (50 kDa) [12], secreted by vascular endothelial cells and is involved in the regulation of important endothelial functions such as cell adhesion, proliferation, migration, and neovascularization [13]. Endocan secretion increases in a variety of endothelium-related pathological conditions such as atherosclerosis, inflammation, infections, and tumor progression [13, 14].

Nesfatin-1, a 82 amino acid polypeptide, was first discovered in 2006, found to be expressed in the hypothalamus, and involved in regulating food intake [15, 16]. Nesfatin-1 has been found to have some cardiovascular effects such as regulation of blood pressure and heart rate, role in cardiomyocyte metabolism, and protection against ischemia/reperfusion injury [17].

The aim of our study was to evaluate the clinical relevance of serum endocan and nesfatin-1 levels, EAT thickness, and CIMT as markers of increased cardiovascular disease (CVD) risk in patients with PHPT.

## 2. Materials and methods

In this case-control study, 44 patients with clinical evidence of PHPT, i.e. those with hypercalcemia and high or inappropriately normal PTH levels, and 40 healthy control subjects were enrolled from October 2019 to October 2020.

Study participants were selected from patients diagnosed at the Department of Endocrinology and Metabolism at Necmettin Erbakan University Meram Medical School. Necmettin Erbakan University Ethics Committee approved the study with the approval no 2019/2092 and the date 20.09.2019. The study complied with the Ethical Principles of the World Medical Association Declaration of Helsinki. Written “Voluntary Informed Consent Form” was obtained from the patient and the control group who agreed to participate in the study. Demographic and clinical information was collected regarding patients’ sex, age, and biochemical examination.

The participants were recruited from PHPT patients firstly admitted to our department and from healthy volunteers who were admitted for a routine medical assessment. All participants were evaluated by medical history (including smoking status, medications used, HT history), physical examination (weight, height, body mass index as kg/m^2^, arterial blood pressure, waist and hip circumferences) and laboratory measurements (calcium, albumin, phosphorous, PTH, HbA_1c_, fasting glucose, lipids, complete blood count, urea, creatinine). Subjects with clinical evidence of CVD (coronary, peripheral, carotid artery), any other major disease (hepatic failure, end-stage renal failure, any suspicious renal disease, any malignancy, autoimmune diseases, acute or chronic infections), DM, recent history of trauma or surgery, or pregnancy were excluded. In addition, those younger than 18 years old and those over 65 years old were excluded from the study. Demographic data of the groups are shown in Table 1. Control group (n = 40) was age-, sex-, and body-mass-index matched with the PHPT group (n = 44).

Ten milliliters of peripheral venous blood samples were obtained from all patients and sent to the laboratory for processing. After centrifugation at 4000 rpm for 10 min at 4 °C (Hitachi Koki^®^, Tokyo, Japan), plasma aliquots were stored at −80 °C (Panasonic MDF-U56VC-PA, Mexico City, Mexico) until analysis.

Serum concentrations of endocan were measured by an ELISA reader (Power Wave XS; Bio-Tek, Massachusetts, USA) and ELISA kit (Human ESM1 ELISA kit, E-EL-H1557, Elabscience, TX, USA) according to the manufacturer’s standard protocol. The endocan levels were expressed as pg/mL [18]. Serum concentrations of nesfatin-1 were measured by an ELISA reader (Power Wave XS; Bio-Tek, Massachusetts, USA) and ELISA kit ((Human ESM1 ELISA kit, E-EL-H2373, Elabscience, TX, USA)) according to the manufacturer’s standard protocol. The nesfatin-1 levels were expressed as pg/mL.

### 2.1. CIMT assessment

CIMT measurements were performed by an experienced cardiologist, blinded to patients’ clinical and laboratory data, using a commercially available ultrasound system with a 2.5–3.5 MHz transducer (iE33, xMATRIX Echocardiography System, Phillips Medical System, Bothell, WA, USA), according to the consensus guidelines for carotid ultrasound for CVD risk assessment as described previously [19]. Ultrasound scans were performed with the patients lying in a supine position with the head resting comfortably and the neck slightly hyperextended and rotated in the opposite direction of the probe. Three CIMT values were obtained in three sites, respectively, which included the common carotid artery, the carotid bulb (1 cm distally), and the internal carotid artery. The values were measured within a 1 cm length at each site. The mean of 18 repetitive measurements was documented (nine for each carotid artery) and defined as mean CIMT. Maximum CIMT was defined as the mean of the two maximum values, one on each carotid artery.

### 2.2. EAT thickness assessment

After detailed medical history and physical examination, echocardiography of patients were performed by an experienced cardiologist, blinded to patients’ clinical and laboratory data, using a commercially available ultrasound system with a 2.5–3.5 MHz transducer (iE33, xMATRIX Echocardiography System, Phillips Medical System, Bothell, WA, USA). A previously validated method was used to assess EAT thickness in captured images [20]. Briefly, EAT thickness was measured on the free wall of right ventricle from the parasternal long-axis view. The aortic annulus was used as the reference point. Echo-free space between the echo-dense pericardial layers on two-dimensional echocardiography was measured perpendicularly on the ahead of the right ventricle free wall at the end of diastole. After the measurement of two beats, maximum EAT thickness values were measured and the average value was obtained. This method was shown to be strongly correlated with various metabolic markers.

### 2.3. Statistical analysis

Statistical analysis was performed using SPSS 21.0 program. Continuous variables were given as mean ± standard deviation if the distribution was normal, and as median (minimum–maximum) if the distribution was not normal. The Kolmogorov–Smirnov test was used to determine whether the data were normally distributed or not. In the comparison of independent group differences, the significance test of the difference between the two means (independent samples t-test) was used when the parametric test assumptions are provided; The Mann–Whitney U test was used to compare the independent group differences when the parametric test assumptions were not provided. Chi-squared test was used to compare categorical variables between independent groups. The Pearson test was used for correlation analysis between numerical variables with normal distribution, and The Spearman test was used for correlation analysis between numerical variables that did not show normal distribution. For differences, p < 0.05 was considered statistically significant.

## 3. Results

The study involved 84 cases, including 44 PHPT patients and 40 healthy controls. Of the 44 PHPT patients, 39 (88.6%) were female and 5 (11.4%) were male. Of the 40 control subjects, 36 (90%) were female and 4 (10%) were male. The mean age of the PHPT patients was 49.95 ± 10.55 years, the mean age of the control subjects was 48.42 ± 10.19, and there was no difference between the two groups in terms of age and sex (Table 1).

Waist circumference, hip circumference, waist/hip ratio, low-density lipoprotein (LDL) cholesterol level, and CIMT values were statistically significantly higher in the PHPT group compared to the control group. In addition, as expected, calcium, PTH, and alkaline phosphatase levels were higher and phosphorus levels were lower in PHPT patients than in the control group.

Body mass index, systolic blood pressure (SBP), diastolic blood pressure (DBP), urea, creatinine, triglyceride, magnesium, ejection fraction values were higher in the PHPT group compared to the control group, but there was no statistically significant difference. Glucose, HbA1c, high-density lipoprotein (HDL) cholesterol, albumin, C-reactive protein, vitamin D, thyroid-stimulating hormone, and EAT thickness values were higher in the control group compared to the PHPT group, but there was no statistically significant difference.

Serum endocan level was measured as 824.8 ± 351 pg/mL and 826.68 ± 373.65 pg/mL in PHPT patients and control group, respectively. Serum nesfatin-1 level was measured as 148.8 ± 4.5pg/mL and 149.14 ± 5.66 pg/mL in the PHPT patients and the control group, respectively. There was no difference between the two groups in terms of serum endocan and nesfatin-1 levels (p = 0.963 and p = 0.510, respectively) (Table 1 and Figure).

In correlation analysis, a negative correlation was found between PTH and LDL cholesterol levels (p = 0.001). No significant relationship was found between other parameters (Table 2).

## 4. Discussion

As a result of our study, we found that CIMT, LDL cholesterol, waist circumference, hip circumference, and waist/hip ratio values were higher in the mild PHPT patients. We found that there was no difference between the PHPT patients and the normal population in terms of other parameters such as endocan, nesfatin-1, and EAT thickness.

High calcium and PTH levels in PHPT patients have been shown to negatively affect vascular function, especially by disrupting vasodilation [21]. CIMT is often used as an indicator of vascular dysfunction, subclinical atherosclerosis, and increased cardiovascular risk. There are studies in the literature reporting conflicting results about the effects of PHPT on CIMT, and some of these studies reported increased CIMT in patients with PHPT [22 – 26]. Nuzzo et al. showed that PHPT caused an increase in CIMT values in 20 patients with calcium levels between 11.4 mg/dL and 13.5 mg/dL [22]. Walker et al. showed that PHPT caused an increase in CIMT in patients with calcium levels of 10.5 ± 0.5 mg/dL, albeit with lower calcium levels than the study of Nuzzo et al. [22, 23]. More remarkably, Karakose et al. evaluated 48 patients with newly diagnosed PHPT before and six months after parathyroidectomy, and they found that postoperative CIMT values were significantly lower than preoperative values [25]. Cansu et al. investigated the effect of parathyroidectomy by grouping patients with PHPT according to whether they are hypercalcemic or normocalcemic, and the authors reported that parathyroidectomy reduced CIMT in patients with hypercalcemic PHPT, but had no effect on CIMT in patients with normocalcemic PHPT [26]. On the other hand, in some of the studies, no significant CIMT increase was found in PHPT patients [27 – 31]. In a study, it was shown that there was no increase in CIMT in PHPT patients without classically determined cardiovascular risk factors (e.g., diabetes, hyperlipidemia, hypertension, obesity, smoking habit), whereas PHPT patients with risk factors had increased CIMT [24].

Not only bone and kidneys, but also cardiomyocytes, endothelial cells, and smooth muscle cells express PTH receptors [32]. In some experimental studies, it has been shown that PTH can selectively target the vessel wall by acting on specific receptors on cardiomyocytes, endothelial cells, and smooth muscle cells, and it is thought to play a role in the pathophysiology of PTH-mediated structural and functional cardiovascular changes [33–35]. In our study, we found that CIMT was increased in mild PHPT patients without known classical CVD risk factors. We think that this result is associated with increased PTH levels in PHPT patients.

Adipose tissue is considered an endocrine organ that secretes many hormones and adipokines, including leptin, adiponectin, and tumor necrosis factor-α (TNF-α), which have proinflammatory and proatherogenic effects and lead to negative metabolic and cardiovascular consequences [36, 37]. Epicardial adipose tissue synthesizes and secretes adipokines and bioactive factors that can reach the myocardium via vasocrine and/or paracrine pathways, thereby adversely affecting coronary circulation and leading to myocardial dysfunction and hypertrophy, coronary artery disease, and heart failure [38]. There are a limited number of studies investigating EAT thickness in PHPT patients. In our study, the EAT thickness of PHPT patients were found to be similar to the general population. There are also studies in the literature reporting that EAT thickness is increased in patients with PHPT, unlike our study, and in one of these studies, it was reported that EAT thickness was correlated with serum PTH level and hypercalcemia [39]. In light of previous studies, we think that the increased PTH level in the PHPT patients is the factor leading to increased EAT thickness and thus increased CVD risk. The most likely reason why we did not find an increase in EAT thickness in patients with PHPT is the small sample size.

Classically known cardiovascular risk factors include DM, hyperlipidemia, HT etc. In our study, no significant difference was found between PHPT patients and healthy controls in terms of glucose, SBP, DBP, triglyceride, HDL cholesterol levels. We found that LDL cholesterol levels were lower in PHPT patients than in the control group. There are conflicting data in this context in the literature. In a study investigating CVD risk factors in PHPT patients, high SBP, fasting glucose, insulin, and uric acid levels were found. In the same study, it was reported that SBP, DBP, triglyceride, fasting glucose, insulin, and uric acid levels were higher in severe PHPT patients, and SBP and insulin levels were higher in mild PHPT patients [4]. In another study conducted with mild PHPT patients, SBP and triglyceride levels were found to be higher [6]. In another study investigating CVD risk factors in patients with mild PHPT, no relationship was found in terms of classical risk factors such as DM, hyperlipidemia, and HT [40].

Advances in biomarker research and recent developments in the field of CVD have led to greater emphasis on more sensitive screening methods and early detection, and improved treatments that result in more positive clinical outcomes [41]. Endocan is secreted by vascular endothelial cells and is known as a marker of endothelial dysfunction. It has been shown in some studies that the high endocan level may be closely related to the development and progression of CVD [42 – 44]. Nesfatin-1 is a new neuropeptide with metabolic regulatory effects. Some studies have found that Nesfatin-1 is associated with CVD [45 – 46]. The current study is important as it is the first study to evaluate whether the increased risk of CVD in PHPT patients can be demonstrated by biochemical markers such as endocan and nesfatin-1. In our study, which is a first in this field, no significant difference was found between PHPT patients and healthy controls in terms of serum endocan and nesfatin-1 levels. We think that the fact that endocan and nesfatin-1 levels were found to be similar to the control group in patients with PHPT in our study may be due to the small sample size. In our study, CIMT, which is a marker of CVD risk, was found to be increased in mild PHPT patients, but no relationship was found in terms of serum endocan and/or nesfatin-1 levels.

In the current study, no significant correlation was found between PTH and CIMT and EAT. However, we found a negative correlation between PTH and LDL cholesterol. We think that the negative correlation between PTH and LDL cholesterol is coincidental due to the small sample size.

Some limitations of our study are the small sample size, especially when it comes to men, and the fact that the majority of the patient group consists of patients with slightly elevated calcium levels. In addition, the fact that parameters such as CIMT and EAT were examined instead of more sensitive and specific methods such as coronary calcium scoring as an indicator of CVD risk and that no evaluation was made after surgical and/or medical treatment are among the important limitations of the current study. One of the limitations of our study is that the control group selection was made from healthy controls admitted to the hospital for different reasons.

In conclusion, we found that CIMT was increased in patients with mild PHPT, and we, therefore, think that CVD risk may be higher in these patients. Studies with higher number of patients and longer follow-up are needed to determine the factors leading to increased CVD risk in patients with PHPT.

## Figures and Tables

**Figure:**
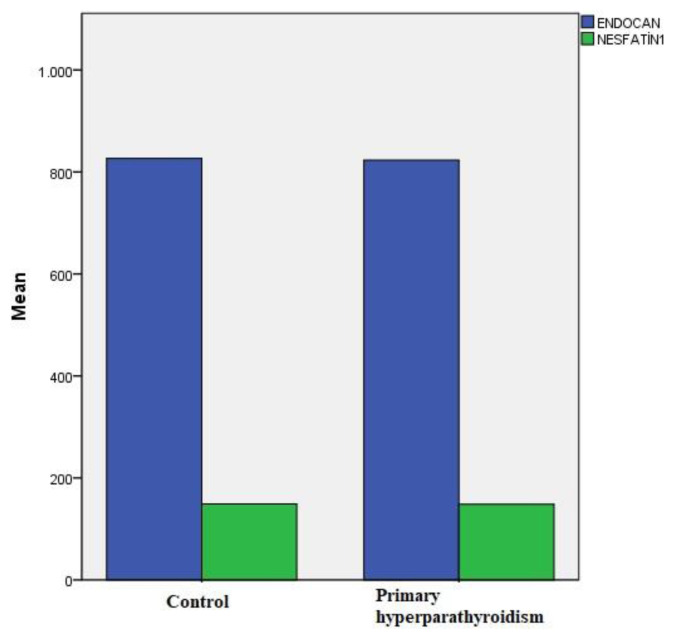
Endocan and nesfatin-1 levels (pg/mL) in PHPT patients and controls.

**Table 1:** Demographical and laboratory parameters of study population.

Variables	Study population (n = 84)	Control group (n = 40)	PHPT group (n = 44)	p-value
Age (years)	48.42 ± 10.19	46.73 ± 9.62	49.95 ± 10.55	0.148
Female, n (%)	75 (89)	36 (90)	39 (88.6)	0.858
BMI (kg/m^2^)	30.15 ± 5.99	29.63 ± 6.44	30.62 ± 5.58	0.456
SBP (mmHg)	120.71 ± 20.03	116.25 ± 19	124.77 ± 20.28	0.051
DBP (mmHg)	78.63 ± 13.24	76.62 ± 12.31	80.45 ± 13.92	0.185
WC (cm)	89.83 ± 13.91	82.62 ± 11.76	96.38 ± 12.49	0.001
HC (cm)	106.4 ± 13.75	100.4 ± 11.91	111.86 ± 13.14	0.001
Waist/hip ratio	0.84 ± 0.06	0.82 ± 0.05	0.86 ± 0.07	0.005
**Laboratory parameters**				
Urea (mg/dL)	25.76 ± 7.65	25.33 ± 6.81	25.79 ± 7.78	0.921
Creatinine (mg/dL)	0.78 ± 0.59	0.73 ± 0.14	0.82 ± 0.81	0.501
Glucose (mg/dL)	94.11 ± 10.05	94.9 ± 9.8	93.39 ± 10.33	0.497
HbA1C (%)	5.57 ± 0.36	5.58 ± 0.36	5.56 ± 0.35	0.997
LDL-C (mg/dL)	119.86 ± 36.56	111.24 ± 36.72	127.87 ± 34.94	0.038
HDL-C (mg/dL)	52.77 ± 16.95	53.27 ± 14.22	52.31 ± 19.31	0.799
Triglyceride (mg/dL)	160.47 ± 94.34	140.12 ± 93.77	179.39 ± 91.94	0.058
Calcium (mg/dL)	10.41 ± 1.06	9.48 ± 0.33	11.26 ± 0.73	0.001
Phosphorus (mg/dL)	3.44 (1.38–41)	4.62 (1.9–41)	2.54 (1.39–3.69)	0.036
Albumin (g/dL)	4.53 ± 2.94	4.54 ± 2.51	4.53 ± 3.26	0.849
ALP (U/L)	124 (43–870)	71 (64–84)	128 (43–870)	0.005
Magnesium (mg/dL)	2.02 ± 0.21	1.85 ± 0.07	2.03 ± 0.20	0.220
CRP (mg/L)	4.26 (0.11–26)	5.28 (0.17–26)	3.26 (0.18–18)	0.062
Endocan (pg/mL)	824.8 ± 351	826.68 ± 373.65	823.09 ± 333.94	0.963
Nesfatin-1 (pg/mL)	148.8 ± 4.5	149.14 ± 5.66	148.49 ± 3.12	0.510
Vitamin D (ug/L)	14.75 ± 8.38	18.3 ± 14.97	14.35 ± 7.48	0.324
PTH (ng/L)	138 (25–1375)	34 (25–42)	202 (72–1375)	0.001
TSH (mU/L)	2.01 ± 1.22	2.06 ± 1.19	1.96 ± 1.26	0.725
**Echocardiographical parameters**				
CIMT (cm)	0.05 ± 0.01	0.049 ± 0.01	0.058 ± 0.01	0.001
EAT thickness (cm)	0.36 ± 0.11	0.375 ± 0.12	0.35 ± 0.11	0.454
EF (%)	60.49 ± 1.8	60 ± 1.24	60.5 ± 1.82	0.788

All values are presented as mean value ± SD, median value (minimum–maximum) or n (%). ALP: alkaline phosphatase, BMI: body mass index, CIMT: carotid intima media thickness, CRP: C-reactive protein, DBP: diastolic blood pressure, EAT: epicardial adipose tissue, EF: ejection fraction, HC: hip circumference, HDL-C: high-density lipoprotein cholesterol, LDL-C: low-density lipoprotein cholesterol, PHPT: primary hyperparathyroidism, PTH: parathyroid hormone, SBP: systolic blood pressure, TSH: thyroid-stimulating hormone, WC: waist circumference

**Table 2:** Correlation of parathyroid hormone with metabolic and biochemical parameters.

Variables	r-value	p-value
Endocan	−0.039	0.802
Nesfatin-1	−0.021	0.989
CIMT	0.089	0.555
EAT thickness	−0.145	0.337
Vitamin D	−0.317	0.035
BMI	−0.176	0.235
Waist/hip ratio	0.056	0.723
Calcium	0.482	0.001
Phosphorus	−0.496	0.001
LDL-C	−0.512	0.001
CRP	−0.154	0.324

BMI: body mass index, CIMT: carotid intima media thickness, CRP: C-reactive protein, EAT: epicardial adipose tissue, LDL-C: low-density lipoprotein cholesterol.

r indicates Pearson correlation coefficient
